# Binding of Natural and Synthetic Polyphenols to Human Dihydrofolate Reductase

**DOI:** 10.3390/ijms10125398

**Published:** 2009-12-18

**Authors:** Luís Sánchez-del-Campo, Magalí Sáez-Ayala, Soledad Chazarra, Juan Cabezas-Herrera, José Neptuno Rodríguez-López

**Affiliations:** 1 Departamento de Bioquímica y Biología Molecular A, Facultad de Biología, Universidad de Murcia, E-30100 Espinardo, Murcia, Spain; 2 Servicio de Análisis Clínicos, Hospital Universitario Virgen de la Arrixaca, Murcia, Spain

**Keywords:** polyphenols, tea catechins, flavonoids, dihydrofolate reductase, antifolates, enzyme inhibition

## Abstract

Dihydrofolate reductase (DHFR) is the subject of intensive investigation since it appears to be the primary target enzyme for antifolate drugs. Fluorescence quenching experiments show that the ester bond-containing tea polyphenols (-)-epigallocatechin gallate (EGCG) and (-)-epicatechin gallate (ECG) are potent inhibitors of DHFR with dissociation constants (*K_D_)*of 0.9 and 1.8 μM, respectively, while polyphenols lacking the ester bound gallate moiety [e.g., (-)-epigallocatechin (EGC) and (-)-epicatechin (EC)] did not bind to this enzyme. To avoid stability and bioavailability problems associated with tea catechins we synthesized a methylated derivative of ECG (3-*O*-(3,4,5-trimethoxybenzoyl)-(-)-epicatechin; TMECG), which effectively binds to DHFR (*K_D_* = 2.1 μM). In alkaline solution, TMECG generates a stable quinone methide product that strongly binds to the enzyme with a *K_D_* of 8.2 nM. Quercetin glucuronides also bind to DHFR but its effective binding was highly dependent of the sugar residue, with quercetin-3-xyloside being the stronger inhibitor of the enzyme with a *K_D_* of 0.6 μM. The finding that natural polyphenols are good inhibitors of human DHFR could explain the epidemiological data on their prophylactic effects for certain forms of cancer and open a possibility for the use of natural and synthetic polyphenols in cancer chemotherapy.

## 1. Introduction

Recent studies have presented data that show a variety of biological activities of tea catechins, compounds which constitute about 15% (dry weight) of green tea [1,2]. Green tea catechins include: (-)-epigallocatechin gallate (EGCG), (-)-epigallocatechin (EGC), (-)-epicatechin gallate (ECG) and (-)-epicatechin (EC). EGCG is the most abundant (one 240 mL cup of brewed tea contains up to 200 mg EGCG), and many health benefits, including antioxidant, antibiotic and antiviral activities, have been attributed to this compound [3–5]. Some authors consider EGCG alone to be the active anticancer component, while others suggest that other tea constituents also have antiproliferative or anticarcinogenic properties [6]. Green tea extracts have been shown *in vitro* to stimulate apoptosis of various cancer cell lines, including prostate, lymphoma, colon, and lung [1,6–8]. Moreover, EGCG was reported to inhibit tumour invasion and angiogenesis, processes that are essential for tumour growth and metastasis [6]. Despite great efforts during the last two decades to understand the anticarcinogenic activity of tea, the exact mechanism(s) of action are not well defined. Therefore, deciphering the molecular mechanism by which green tea or its polyphenols impart their antiproliferative effects could be important and may result in improved opportunities for the treatment of cancer.

**Figure 1.**
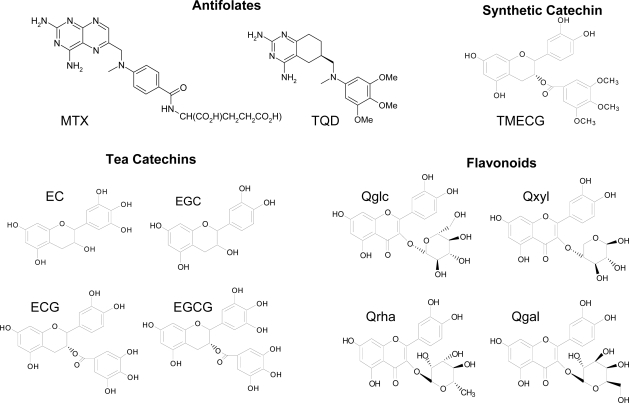
Chemical structures of classical and non-classical antifolates compared with natural and synthetic polyphenols. Abbreviations: MTX, methotrexate; TQD, (R)-6-{[methyl-(3,4,5-trimethoxyphenyl)-amino]methyl}-5,6,7,8-tetrahydroquinazoline-2,4-diamine; EC, (-)-epicatechin; EGC, (-)-epigallocatechin; ECG, (-)-epicatechin gallate; EGCG, (-)-epigallocatechin gallate; TMECG, 3-O-(3,4,5-trimethoxybenzoyl)-(-)-epicatechin; Qglc, quercetin-3-β-D-glucoside; Qxyl, quercetin-3-D-xyloside; Qrha, quercetin-3-rhamnoside; Qgal, quercetin-3-D-galactoside.

**Figure 2.**
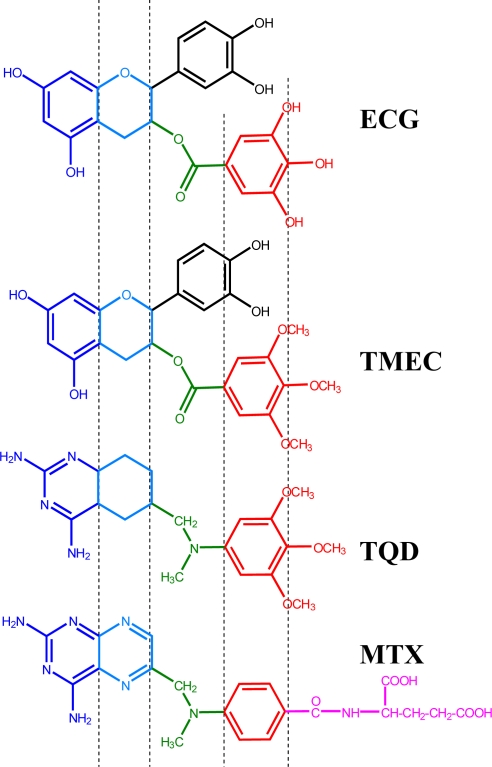
Structural comparison of natural (ECG) and synthetic (TMECG) polyphenols with classical (MTX) and non-classical (TQD) antifolates.

Dihydrofolate reductase (DHFR; 5,6,7,8-tetrahydrofolate: NADP^+^ oxidoreductase, EC 1.5.1.3) catalyzes the reduction of 7,8-dihydrofolate (DHF) to 5,6,7,8-tetrahydrofolate (THF) in the presence of coenzyme NADPH as follows: DHF + NADPH + H^+^ → THF + NADP^+^. This enzyme is necessary for maintaining intracellular pools of THF and its derivatives which are essential cofactors in one-carbon metabolism. Coupled with thymidylate synthase [9], it is directly involved in thymidylate (dTMP) production through a *de novo* pathway. DHFR is therefore pivotal in providing purines and pyrimidine precursors for the biosynthesis of DNA, RNA and amino acids. In addition, it is the target enzyme [10] for antifolate drugs such as the antineoplastic drug methotrexate (MTX) and the antibacterial drug trimethoprim (TMP). Because of its biological and pharmacological importance, DHFR has been the subject of extensive structural and kinetic studies [11]. Based on the observation that classical (MTX) and non-classical (TMP or TQD) antifolate compounds possess similar chemical structures to some tea polyphenols [12] (Figures 1 and 2), we started to work on the hypothesis that tea catechins could inhibit DHFR activity. Suppression of DNA synthesis by tea catechins could explain many of the observed effects on cancer inhibition by these compounds. Recently, we have shown that ester bonded gallate catechins isolated from green tea, such as EGCG and ECG are potent inhibitors of DHFR activity *in vitro* at concentrations found in the serum and tissues of green tea drinkers (0.1–1.0 μM) [12]. EGCG exhibited the kinetic characteristics of a slow-binding inhibitor of DHF reduction with bovine liver DHFR but of a classical, reversible, competitive inhibitor with chicken liver DHFR. Structural modelling showed that EGCG can bind to human DHFR in a similar orientation to that observed for a number of structurally characterized DHFR inhibitor complexes [12]. These results suggested that EGCG could act as an antifolate compound in the same way as MTX and TMP. In the present work, we have studied the binding of several natural and synthetic polyphenols to human DHFR and the structural requirements for such binding have been analyzed.

## 2. Results and Discussion

### 2.1. Structural Requirements for the Binding of Tea Polyphenols to Human DHFR

**Figure 3.**
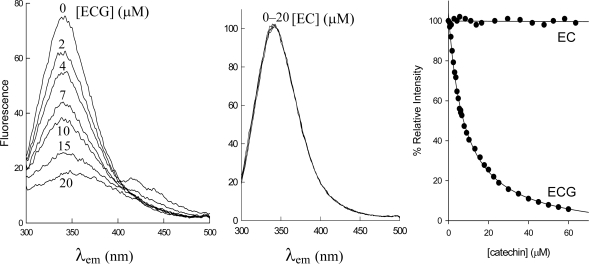
Titration fluorescence experiments for the binding of ECG and EC to human DHFR. In the left panel points are experimental (after correction for enzyme dilution) and the lines are best-fit theoretical curves. The enzyme concentration was 0.1 μM.

Green tea extracts containing significant amounts of tea catechins strongly inhibited the activity of bovine liver DHFR [13]. In order to detect which components of these extracts were responsible for such inhibition, human DHFR activity was assayed in the presence of EC, EGC, ECG or EGCG. The results showed that both ECG and EGCG were potent inhibitors of the human enzyme, while polyphenols lacking the ester bound gallate moiety (e.g., EGC and EC) did not inhibit this enzyme. The effective binding of ECG to free human DHFR was determined by following the decrease in enzyme fluorescence that occurs after formation of the enzyme-inhibitor complex (Figure 3). When DHFR fluorescence is excited at 290 nm its emission spectrum shows a maximum at 340–350 nm. The binding of ECG quenches this fluorescence and the data showed that the dissociation constant of the enzyme-inhibitor complex was 1.78 ± 0.1 μM (Table 1).

**Figure 4.**
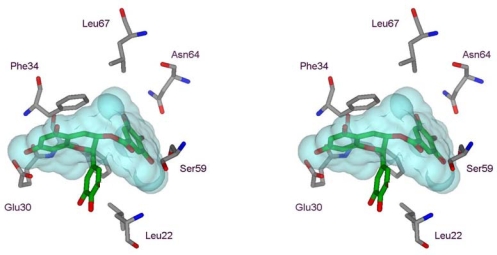
Wall-eyed stereo view of ECG modeled into the folate-binding site of human DHFR. Carbon atoms of the ECG ligand and surrounding protein are colored green and grey respectively. Residue Phe-31, located behind the ECG, is unlabelled. Four different ligands from human and chicken DHFR crystal structures were used to define a binding envelope, shown in cyan; these were placed in a common orientation by superimposing backbone atoms from a common set of protein residues located around the ligands. Ligands from the following PDB structure files were used; 1DR1 (biopterin), 1S3V (TQD), 1S3W, and 1DLR. The figure was prepared using ViewerLite software.

Similar results were obtained for EGCG (Table 1); however, EC did not modify the emission spectra of human DHFR (Figure 3). These results indicate that the ester-bound gallate moiety is essential for the inhibition of this enzyme, as has been determined in the case of the bovine enzyme [12,13]. On searching the available ligand-bound human DHFR structures in the Protein Data Bank (PDB) [14], we identified a 1.8 Å structure (PDB accession code 1S3V [15]) containing a tetrahydroquinazoline antifolate ligand, TQD (Figures 1 and 2), as the best available structural match for ECG. Using the position of TQD as a guide, ECG was docked into this protein structure and the ECG-protein composite was then energy minimized using Insight II software (Figure 4). Comparison with a range of other DHFR structures containing folate or various inhibitors showed that most of the ECG lies within the consensual substrate/inhibitor envelope, with the exception of the non-ester trihydroxybenzene moiety. In order to accommodate this ring, the Leu-22 sidechain is required to adopt a different orientation; a precedent for this movement is provided by the crystal structure of a Tyr-22 mutant, which displays a similar geometry at this residue [16]. Although folate, TQD, MTX and ECG are significantly different in terms of their structural formulae, they have similar 3D shapes, and this appears to be an important determinant of their binding to DHFR. There are also specific hydrogen bonding interactions, most notably that involving Glu-30. For folate and MTX, adjacent heterocyclic and amino nitrogens of the ligand form a pair of hydrogen bonds with the two oxygens of the Glu-30 sidechain (both O···N distances ≤ 2.8 Å). In contrast, ECG has only a single phenolic OH group available for hydrogen bonding to Glu-30 (O···O distance 2.7 Å).

### 2.2. Binding of Synthetic Catechins to Human DHFR

**Figure 5.**
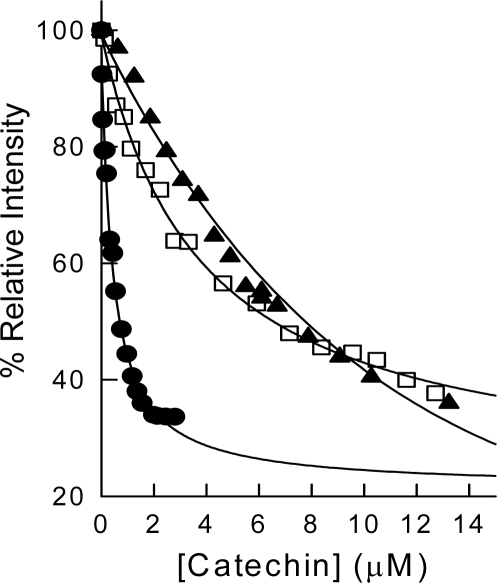
Titration fluorescence experiments for the binding of TMECG (□) and TMECG-QM (•) to human DHFR. Points are experimental (after correction for enzyme dilution) and the lines are best-fit theoretical curves. The enzyme concentration was 0.1 μM. For comparison the data obtained for ECG (▴) binding to human DHFR is included in the figure.

**Figure 6.**
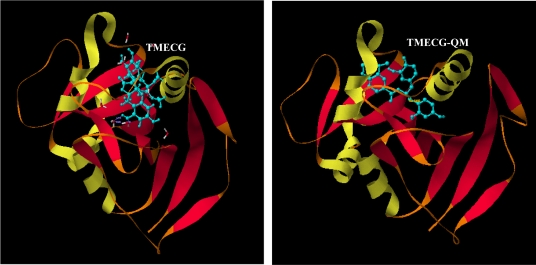

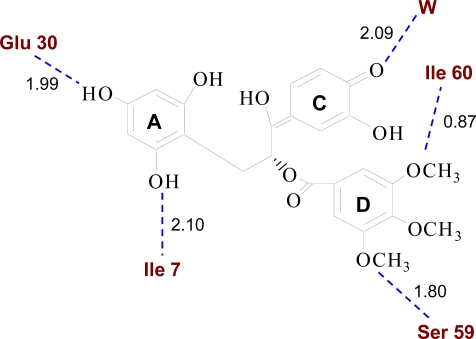
Molecular modelling for the binding of TMECG and TMECG-QM to human DHFR. The protein is depicted in ribbon representation and colored by secondary structures (*i.e.*, helix, strand, and loop). The lower panel represents the proposed structure for TMECG-QM and the hydrogen-bonding network of TMECG-QM in human DHFR. Residues hydrogen bonded directly to TMECG-QM are depicted in red and the predicted distances in Å are showed. W represents a water molecule.

Despite the excellent anticancer properties of tea catechins, they have at least one great limitation: their poor bioavailability. The reduced availability of catechins is related with their low stability in neutral or slightly alkaline solutions and their poor ability to cross cellular membranes [17]. In an attempt to solve such bioavailability problems, we synthesized a 3,4,5-trimethoxybenzoyl analogue of ECG (3-*O*-(3,4,5-trimethoxybenzoyl)-(-)-epicatechin; TMECG; Figure 1), which showed high bioavailability *in vitro* and significant antiproliferative activity against different cancer cell lines [18]. This synthetic catechin also bound human DHFR with an effective dissociation constant of 2.1 ± 0.2 μM (Figure 5; Table 1). Molecular modelling experiments showed that TMECG bound to human DHFR in a similar way to that described for ECG, with specific hydrogen bonding interactions, most notably involving Glu-30 (Figure 6). TMECG was highly stable at neutral pH but at alkaline pH rapidly evolves to an orange compound with an intense visible band at 470 nm. By analogy with the oxidation of quercetin in alkaline solution [19], the proposed structure for this compound is depicted in Figure 6 and was identified as a quinone methide (TMECG-QM) with an open structure. The affinity of human DHFR by TMECG-QM was assayed employing fluorescence quenching and the data showed a dissociation constant of 8.2 ± 0.11 nM (Figure 5; Table 1).

Compared with other inhibitors of the enzyme, TMECG-QM showed intermediate affinity between MTX and catechins; thus, TMECG-QM bound more than two-order of magnitude more strongly than EGCG, ECG or TMECG but forty-times less than MTX (Table 1). Molecular modelling helped us to understand the high affinity of human DHFR for TMECG-QM. The open structure of the TMECG-QM increases its molecular flexibility and adopts a different conformation in the active site of human DHFR (Figure 6).

TMECG-QM maintained the hydrogen bond with Glu-30 side chain (O···O distance 1.99 Å), but three new interactions were detected. Thus, the other phenolic group of the ring A of TMECG-QH forms a hydrogen bond with Ile-7, whereas the other two hydrogen bonds were formed between two oxygens of the methoxy groups of ring D and Ser-59 and Ile-60 (Figure 6). This strong interaction between TMECG-QM and different residues of the protein explains the low dissociation constant for the inhibitor-protein complex.

### 2.3. Binding of Natural Flavonoids to Human DHFR

Flavonoids are naturally occurring polyphenolic compounds and have profound pharmacological properties, and a daily intake of flavonoids is associated with a lower risk of cancer [20]. They are most commonly known for their antioxidant activity; however, it is now known that the health benefits they provide against cancer and heart disease are the result of other mechanisms [20]. Between them quercetin (3,3′,4′,5,7-pentahydroxyflavone) is one of the most studied flavonoids [21]. Quercetin has been shown to have diverse biological activities, including antiproliferative and apoptotic effects [22], although the mechanisms are still obscure. Quercetin treatment caused cell cycle arrest either at the G_1_/S or G_2_/M transition, depending on cell type [22,23]. Moreover, quercetin-mediated apoptosis may be related to many factors such as stress proteins, disruption of microtubules, nuclear factor κB, Cox-2, p53, survivin, c-Jun NH_2_-terminal kinase, mitogen-activated protein kinase kinase-extracellular signal regulated kinase, Bcl-2 family proteins, heat shock proteins, DNA topoisomerase II, release of cytochrome c, and activation of caspases [22–24]. Quercetin has been reported to inhibit the growth of various human cancers, including leukemia, breast, esophagus, colon, prostate, nasopharyngeal, endometrial, and lung cancers [22–24]. Quercetin is one of the major dietary flavonoids particularly abundant in fruits and vegetables [25]. Analysis of plasma of volunteers fed with a quercetin-supplemented diet shows that quercetin is mainly circulating as quercetin glucuronides [26]. Most of the biological studies for assessing the properties of quercetin are performed on quercetin (aglycone), and there is limited information available regarding the biological effects of quercetin glucuronides. Therefore, it is imperative to investigate the biological effects of quercetin glucuronides that are more close to the *in vivo* situation.

**Figure 7.**
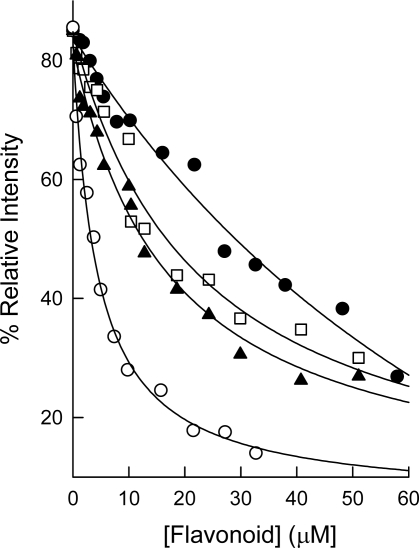
Titration fluorescence experiments for the binding of quercetin glucuronides to human DHFR. Points are experimental (after correction for enzyme dilution) and the lines are best-fit theoretical curves. The enzyme concentration was 0.1 μM. (•) Qglc (□) Qgal (▴) Qrha (○) Qxyl.

The high structural similarity between quercetin glucuronides and ester bound gallate tea catechins (Figure 1) aim us to study the binding of these compounds to human DHFR. The four quercetin glucuronides used in this study have the same core structure (quercetin) but differ in the sugar moiety and in the number and spatial position of the OH groups linked to the sugar ring. The affinity of DHFR for these compounds will help us to identify the optimal conformation for the interaction and inhibition of human DHFR by these quercetin glucuronides. Figure 7 shows the fluorescence quenching of human DHFR by these flavonoids and Table 1 presents the calculated dissociation constants. Qxyl bound human DHFR much stronger than the other three quercetin glucuronides with a dissociation constant of 0.59 ± 0.08 μM. Qxyl and ECG show similar chemical structure (Figure 1) and similar dissociation constants for their binding to human DHFR (Table 1). However, the presence of a sugar ring in Qxyl instead a highly reactive phenolic gallate moiety in ECG could represent an advantage with respect to its stability and bioavailability, which could improve the effectiveness of Qxyl as an antifolate agent.

### 2.4. Physiological and Therapeuthical Relevance

In addition to the antioxidant activity of natural polyphenols, the finding that some of them are good inhibitors of human DHFR could explain the epidemiological data on the prophylactic effects of diets high in polyphenols for certain forms of cancer. A functional link between chronic inflammation and cancer has long been suspected. This link is of great interest in the context of this study because antifolates have shown remarkable anti-inflammatory activity [27]. Most solid tumours contain many non-malignant cells, including immune cells and blood-vessel cells, which are important in inflammation, although the crucial molecular pathways that permit communication between abnormally growing cancer cells and these inflammatory cells remain unknown. A mouse model of inflammation-associated cancer now implicates the gene transcription factor NF-κB and the inflammatory mediator known as TNF-α in cancer progression [28]. Several of the anti-inflammatory effects of MTX and other antifolates can be explained by the suppression of the activation of NF-κB, a multisubunit factor known to play a role in inflammation, immune modulation and cell proliferation. NF-κB is primarily composed of proteins with molecular masses of 50 kDa (p50) and 65 kDa (p65) and is retained in the cytoplasm by an inhibitory subunit, IκBα. NF-κB is activated by a wide variety of inflammatory stimuli, including TNF-α, which induces the phosphorylation-dependent degradation of IκBα, allowing active NF-κB to translocate to the nucleus and regulate gene expression. Suppression of the activation of NF-κB has been an effect widely observed in model cancer cells treated with tea catechins [29] of quercetin derived flavonoids [22–24]. These polyphenols are abundant in tea and other fruits and vegetables and the data presented here on its antifolate activity indicated that by regular consumption these polyphenols can combat chronic inflammation, and therefore, impede cancer formation. The data presented here indicate that natural and synthetic polyphenols may well be beneficial, not only in the prevention but also in the treatment of cancer. Antifolates are usually used as chemotherapeutic agents for certain types of cancer. Although antifolates such as MTX attack proliferating tissues selectively, they are also toxic to normal cells. Deleterious side effects are seen against tissues that proliferate as part of their normal function; such tissues include intestinal mucosa, hair cells, and components of the immune system. The “soft” character of the polyphenols studied here could be developed for use in the treatment of cancer with significantly reduced side effects compared to those of the DHFR inhibitors currently in use in chemotherapy such as methotrexate. Recently, we observed that normal human melanocytes were highly resistant to TMECG-induced apoptosis at concentrations of the drug which killed melanoma cells by apoptosis [30]. Thus, our conclusions highlight the potential of natural polyphenols and their synthetic derivatives for clinical application as anti-carcinogenic and antibiotic agents and in the treatment of inflammatory disorders.

## 3. Experimental Section

Highly purified tea polyphenols EGCG (>95%), ECG (>98%), EGC (>98%), and EC (>98%) were purchased from Sigma Chemical Co. (Madrid, Spain). Qgal, Qglc, Qrha and Qxyl were form Fluka (Madrid, Spain). TMECG was successfully synthesized (70% recovery) from catechin with the subsequent inversion of the stereochemistry at C-3 and by reaction of with 3,4,5-trimethoxybenzoylchloride (Sigma) [18]. To synthesize TMECG-QM, a TMECG solution (0.5 mM) in phosphate buffered saline (PBS), pH 7.0, was alkalinize at pH 8.5 with NaOH. The reaction was followed spectrophotometrically at 470 nm and, after completion, the sample was neutralized by adding HCl. Recombinant human DHFR was purchased from Sigma and its concentration was determined by MTX titration of enzyme fluorescence [31].

The fluorescence of DHFR is reduced on binding of substrates and inhibitors, and this property may be used as a convenient method for determining both the enzyme concentration and the dissociation constants of enzyme-ligands complexes. Dissociation constants for the binding of polyphenols to free DHFR were determined by fluorescence titration in an automatic-scanning Perkin-Elmer LS50B spectrofluorimeter with 1.0 cm light path cells and equipped with a 150W xenon (XBO) light source. The formation of the binary complex between the enzyme and the ligand was followed by measuring the quenching of tryptophan fluorescence of the enzyme upon addition of microliter volumes of a concentrated stock solution of ligand. Fluorescence emission spectra were recorded when DHFR fluorescence was excited at 290 nm. All measurements were corrected for dilution and the data from the titration curves were fitted as described previously [32,33]. Titrations were performed in a buffer containing 2-(*N*-morpholino)ethanesulfonic acid (Mes, 0.025 M), sodium acetate (0.025 M), tris(hydroxymethyl)aminomethane (Tris 0.05 M), and NaCl (0.1 M), at pH 7.4.

Molecular modeling was carried out using the Discover module of Insight II (Insight II, release 2000.1, Accelrys Ltd. Cambridge, UK). Human DHFR X-ray crystal structure 1S3V [15] was retrieved from the protein data bank [14], and its TQD ligand was used as a template for positioning of the ECG ligand. The composite protein/ECG model was geometry optimized within Insight II using the consistent valence force field and steepest descent algorithm to a derivative of 1.0. The refined model was validated within InsightII using Prostat.

## 4. Conclusions

The results of this study demonstrate the binding capacity of natural and synthetic polyphenols to human DHFR. The results suggest that *in vivo* inhibition of DHFR by polyphenols could be of importance to explain the prophylactic and anticancer properties described for these natural compounds. This study also indicates that natural polyphenols could be used as guide compounds for development of new antifolates.

## Figures and Tables

**Table 1.** Dissociation constants for the binding of natural and synthetic polyphenols to human DHFR.

**Polyphenol**	*K_D_***(***μ****M*)**
*Tea Catechins*	
EC	*n.d.*^A^
EGC	*n.d.*
ECG	1.78 ± 0.10
EGCG	0.92 ± 0.12
*Synthetic Catechins*	
TMECG	2.1 ± 0.20
TMECG-QM	(8.2 ± 0.21) × 10^−3^
*Flavonoids*	
Qglc	102 ± 9
Qxyl	0.59 ± 0.08
Qrha	17.2 ± 2.4
Qgal	22.8 ± 3.2

A *n.d.*: not determined.
